# Fiber Temperature Sensor Based on Vernier Effect and Optical Time Stretching Method

**DOI:** 10.3390/mi13122215

**Published:** 2022-12-14

**Authors:** Weihao Lin, Yuhui Liu, Yibin Liu, Perry Ping Shum, Mang I Vai

**Affiliations:** 1 State Key Laboratory of Analog and Mixed-Signal VLSI, University of Macau, Macau 999078, China; 2 Department of Electrical and Computer Engineering, University of Macau, Macau 999078, China; 3 Department of Electrical and Electronic Engineering, Southern University of Science and Technology, Shenzhen 518055, China

**Keywords:** temperature sensing, cascading Saganc rings, Vernier effect, optical time-stretching effect

## Abstract

A novel method for ultra-sensitive and ultra-fast temperature sensing has been successfully implemented by cascading Saganc rings to generate the Vernier effect and doing the same dispersive fibers to achieve the optical time-stretching effect. This is different from the traditional point fiber sensor demodulated by optical spectrum analyzer (OSA) whose demodulation speed is usually at the second level. The designed system maps the wavelength domain to the time domain through the dispersive fiber, which can realize the ultra-fast temperature monitoring at the nanosecond level. The cascaded Sagnac ring is composed of polarization maintaining fiber (PMF) which is significantly affected by the thermal-optical coefficient. When the temperature changes, the variation is as high as −6.228 nm/°C, which is 8.5 times higher than the sensitivity based on the single Sagnac ring system. Furthermore, through the optical time stretching scheme, the corresponding response sensitivity is increased from 0.997 ns/°C to 7.333 ns/°C, and the magnification is increased 7.4 times with a response speed of 50 MHz.

## 1. Introduction

In the past decade, optical fiber temperature sensor has been developed rapidly, because of its unique advantages such as high sensitivity, small size, light weight and immunity to electromagnetic interference [1,2,3,4,5]. Interferometers with different structures have built micro-nano laboratories on optical fibers to achieve the ultimate pursuit of sensitivity, including the Mach Zehnder interferometer (MZI) [6,7,8,9], Fabry Perot interferometer (FPI) [10,11,12], Michelson interferometer (MI) [13,14,15,16] and the Sagnac ring structure [17,18,19,20]. However, due to the limitation of demodulation mode, the OSA has a slow demodulation speed, which makes it very difficult to realize real-time online temperature monitoring [21,22,23]. In recent years, various methods have been proposed to solve the problem of sensing accuracy caused by light sources and demodulation schemes. For example, optical fiber ring laser cavity replaces broadband light sources is one of the methods, However, OSA still limits the demodulation speed [24,25,26,27]. The other way is to use the beat frequency change of DBR laser to demodulate the change of external parameters in frequency. However, DBR usually requires polishing to improve sensitivity, which will affect the stability of the system [28,29,30].

Time domain demodulation is a new demodulation scheme for fiber sensor, which can quickly respond to changes in ambient temperature. In the initial stage, scientists usually choose fiber Bragg grating to monitor temperature, which has the advantage of good stability. However, due to the characteristics of the grating itself, its response sensitivity is particularly low [31,32,33]. Therefore, some researchers have proposed the scheme of sensing by using an interference structure combined with the time stretching method. Feng et al. proposed to use Fourier mode locked fiber laser to realize time domain conversion to measure temperature change [34], and achieve microsecond level temperature response characteristics. However, the designed laser structure is expensive and complex. Bai et al., through the time stretching effect realized by fiber mode locked laser combined with dispersive fiber, and using MZI structure and polarization maintaining photonic crystal fiber to design Sagnac loop structure to monitor the strain [35,36], successfully achieved picosecond level micro strain response. However, as far as we know, no literature has examined a fiber temperature sensor based on mode locked laser and time stretching method.

In this paper, for the first time, we propose to realize the Vernier effect based on a cascaded Sagnac ring to achieve the response characteristics of an ultra-high sensitivity temperature sensor. At the same time, combined with the time stretching method generated by dispersive fiber, the wavelength domain changes are mapped to the ultra-fast temperature demodulation scheme in the time domain. In the wavelength domain, the temperature sensitivity is up to −6.288 nm/°C, which is 8.5 times larger than the single loop sensitivity. In the time domain, 7.333 ns/°C is achieved, 7.4 times effective amplification is achieved, and the demodulation speed is as fast as 50 MHz. The proposed scheme improves the temperature demodulation speed by several orders of magnitude, and is expected to provide potential help in industrial applications such as cell life detection, analyzing of culture dish temperature change, electronic product work and other rapid temperature change monitoring with real-time temperature detection requirements.

## 2. Experimental Setup and Working Principle

**Figure 1:**
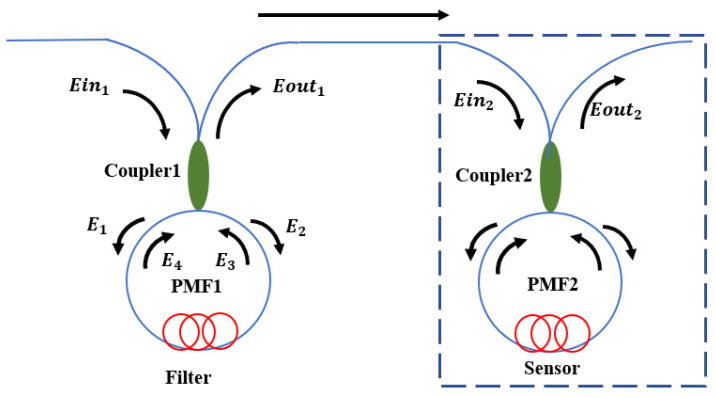
Schematic diagram of the working principle of cascaded Sagnac ring to realize the Vernier effect.

The principle of cascaded Sagnac rings to realize the Vernier effect is shown in Figure 1. The phase difference is caused by the modulation of PMF (PM 1550-125-18/250 YOFC, Wuhan, China) birefringence when light travels clockwise and counterclockwise along the loops. The transmitted electric field intensity can be represented by a Jones matrix [19]:

(1) $$E_{in}=\left[\begin{array}{c} E_{in}^{x}\\ E_{in}^{y} \end{array}\right]$$

A beam of light emitted by the same light source is decomposed into two beams, and they can meet after they travel in the opposite direction for a circle in the same loop, so that interference can be detected in the spectrum. The input electric field is represented by $E_{in}$. $E_{in}^{x}$ and $E_{in}^{y}$ are the two orthogonal polarization components of $E_{in}$. The Jones matrix expression of the 50:50 coupler is [19]:

(2) $$T_{C}=\left[\begin{array}{cc} \sqrt{1-\kappa } & j\sqrt{\kappa }\\ j\sqrt{\kappa } & \sqrt{1-\kappa } \end{array}\right]$$

among which κ represents the coupling coefficient. The electric field output expression after the coupler is [19]:

(3) $$\left[\begin{array}{c} E_{1}\\ E_{2} \end{array}\right]=T_{C}\left[\begin{array}{c} E_{in}\\ 0 \end{array}\right]$$

where $E_{1}$ represents output electric field 1, $E_{2}$ represents light output electric field 2. The Jones matrix of PMF can be expressed as [19]:

(4) $$T_{PMF}=\left[\begin{array}{cc} e^{-j\varphi } & 0\\ 0 & e^{j\varphi } \end{array}\right]$$

(5) $$\varphi =\frac{\pi L\Delta n}{\lambda }$$

among φ is the phase difference of two orthogonal components in the coupler due to the non-uniform circular structure of the fiber core or the residual stress in the manufacturing process, as well as the inherent birefringence in the polarization maintaining fiber, λ is the central wavelength of the input light, L is the effective length of optical fiber inside the whole coupler which equals to 1.45 m and 1.6 m, respectively. We ignored the length of single-mode fiber, since the length of the connected single-mode fiber is about 2.5 cm at the left and right ends. Δn is the refractive index difference between the fast axis and the slow axis in special optical fibers. The electric field intensity expressions $E_{3}$ and $E_{4}$ of the electric field through the coupler can be written as [19]:

(6) $$E_{3}=\left[\begin{array}{cc} e^{-j\varphi } & 0\\ 0 & e^{j\varphi } \end{array}\right]\left[\begin{array}{cc} cos\theta & sin\theta \\ -sin\theta & cos\theta \end{array}\right]E_{1}$$

(7) $$E_{4}=\left[\begin{array}{cc} cos\theta & -sin\theta \\ sin\theta & cos\theta \end{array}\right]\left[\begin{array}{cc} e^{-j\varphi } & 0\\ 0 & e^{j\varphi } \end{array}\right]E_{2}$$

θ indicates the phase shift between two polarization modes when returning to the coupler after one turn of transmission. After passing through the coupler, the expression of the interference spectrum generated can be expressed as [19]:

(8) $$E_{out}=j\sqrt{\kappa }E_{3}+\sqrt{1-\kappa }E_{4\;}$$

**Figure 2:**
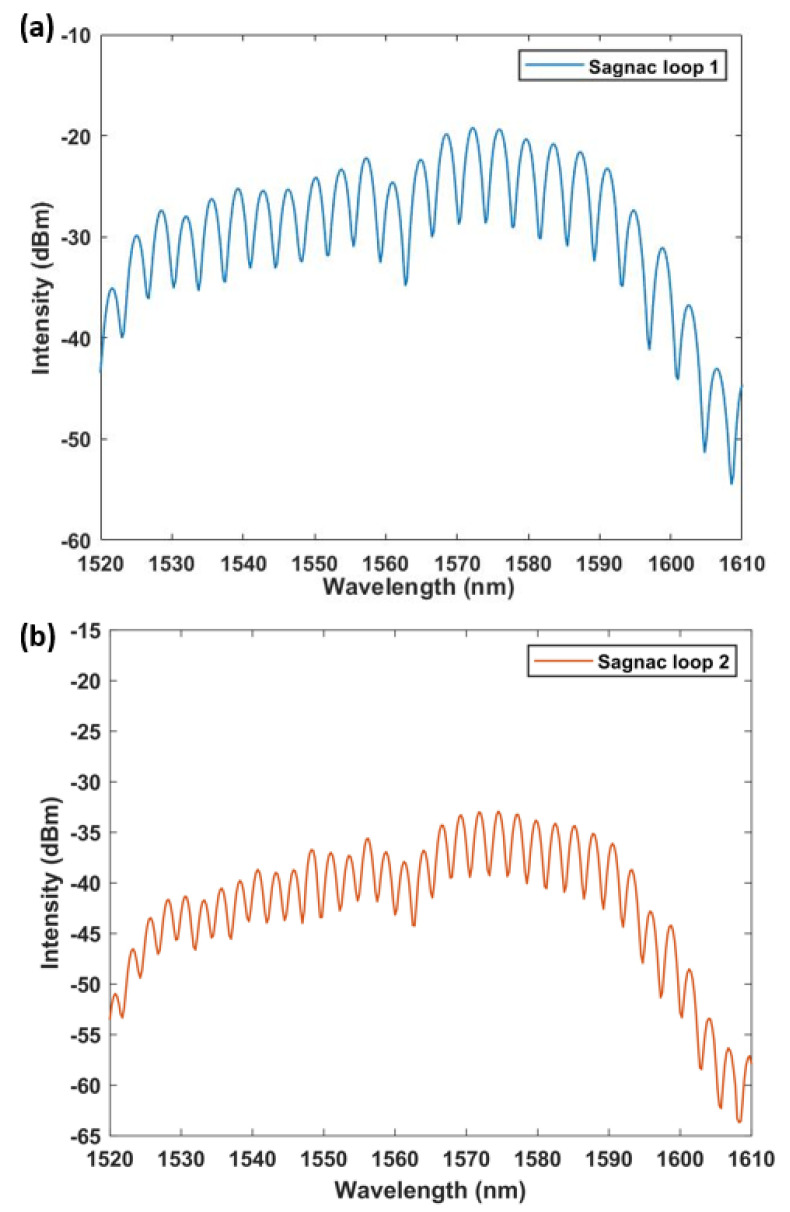
The output spectrum of different Sagnac rings with lengths of (**a**) loop 1 = 1.45 m, (**b**) loop 2 = 1.6 m.

Therefore, we can get the expression of the output spectra in different Sagnac rings. When the PMF length is 1.45 m and 1.6 m, its output spectra are shown in Figure 2. Its free spectral range (FSR) depends on:

(9) $$FSR=\frac{\lambda^{2}}{BL}$$

B represents the birefringence coefficient of the fiber. When the temperature changes, the wavelength offset can be expressed as:

(10) Δλ=Sλ

S is the system’s response to temperature, so the phase change can be expressed as:

(11) $$\varphi =\frac{2\pi BL}{\lambda -S\Delta T}$$

**Figure 3:**
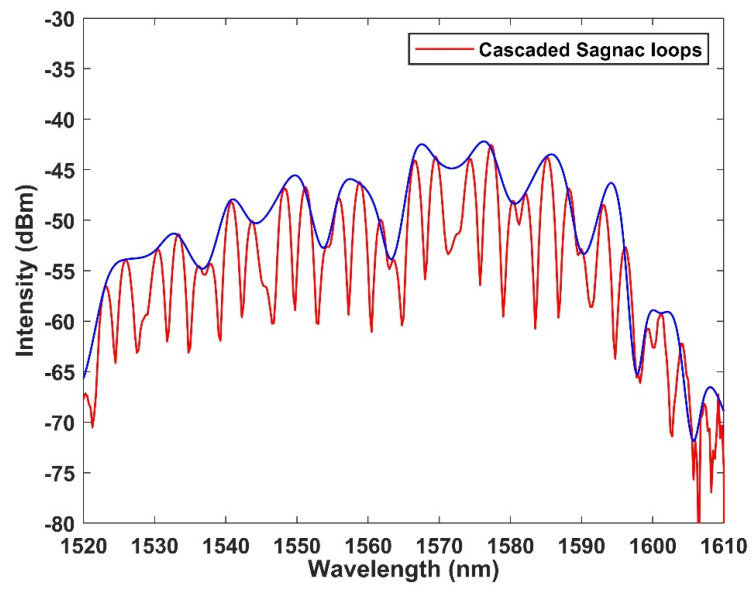
The output spectrum of cascaded Sagnac ring in wavelength domain.

The expression of the output in different Sagnac rings can be found in ref. [37] Vernier effect refers to the large range change of alignment and indexing caused by small measurement value change. When two FSR spectra are very similar to each other, a small change in one of them will cause the entire spectral envelope to move in a large range. By analyzing the initial spectrum and the spectrum after frequency shift, a small amount of change can be amplified, so that measurement sensitivity can be improved in a large range, the output spectrum is shown in Figure 3. The expression of the envelope is [38]:

(12) $$\frac{fsr_{sensor}\times fsr_{filter}}{\left|fsr_{senosr}-fsr_{filter}\right|}$$

According to Formula (12), the magnification is:

(13) $$\frac{fsr_{filter}}{\left|fsr_{senosr}-fsr_{filter}\right|}$$

It is well known that the real-time wavelength to time mapping can be achieved by using devices such as dispersive fiber. The output femtosecond laser is first shaped by the interference spectrum generated by the cascaded Sangac ring. The shaped spectrum is modulated in the time domain through the dispersive fiber. The corresponding formula of the broadened time domain spectrum is as follows:

(14) $$\Delta \lambda =\frac{\Delta t}{D}$$

**Figure 4:**
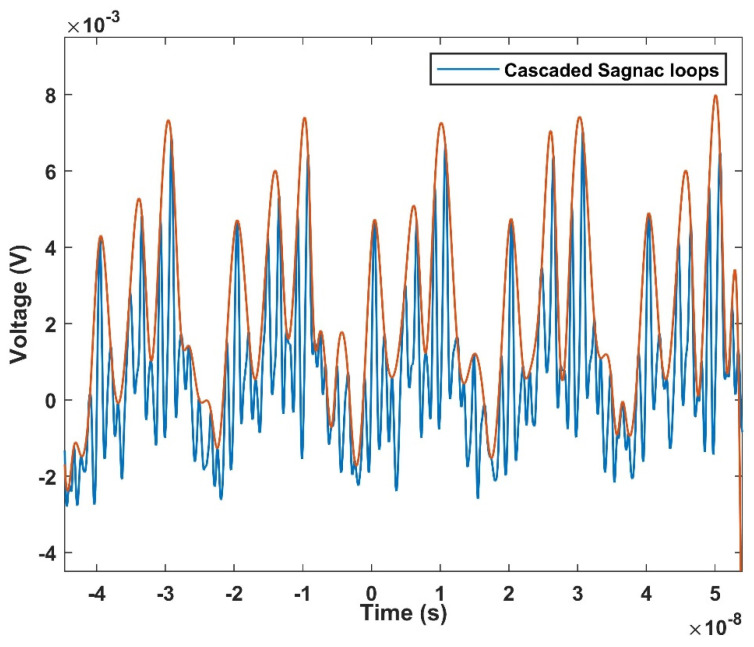
The output spectrum of the cascaded Sagnac ring in time domain.

(15) Δt=DSλ

where Δt is the time shift from the wavelength to the time mapped interference peak in the time domain caused by the change of ambient temperature, which can be recorded by an oscilloscope, and D is the total dispersion coefficient of the dispersive fiber. It illustrates that the time domain spectral shift caused by temperature change is linearly proportional to the temperature change. In addition, the temperature responsivity can be improved by increasing the coefficient of the dispersive fiber. Therefore, the temperature information decoding can simply monitor in real time from the time pulse. The output spectrum is shown in Figure 4. It illustrates that the mapping relationship corresponding to the spectrum shown in Figure 3 is highly consistent.

**Figure 5:**
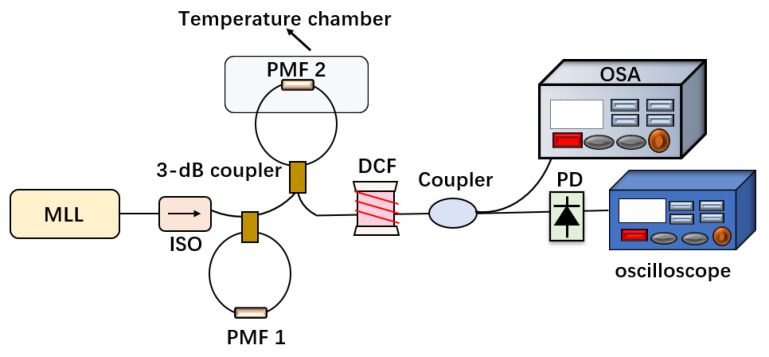
Structure diagram of an ultra-fast temperature detection system based on a time stretching method and the Vernier effect.

The system structure diagram is shown in Figure 5. An ultrafast femtosecond mode-locked fiber laser with an output center wavelength of 1560 nm was selected as the light source, with a repetition rate of 50 MHz and a pulse width of 50 fs (ROI, EFLA-B-1560-50). The output light enters the cascade Saganc loop after passing through the isolator. The isolator is designed to prevent the light source from being damaged by the backscattered light. The loop is composed of two PMF sections with a length of 1.45 m and 1.6 m respectively, which produce the Vernier effect and enhance the detection sensitivity. Next, the modulated pulse light is broadened in the time domain via a dispersive fiber (−497 ps/nm), generating a wavelength to time mapping. Modulated light transmits 70% energy of output light to OSA through a 7:3 coupler, and 30% energy to a 10 GHz photodetector for monitoring and analysis on an oscilloscope (Waverunner 8404 M, 4 GHz, 40 GS/s).

## 3. Results

**Figure 6:**
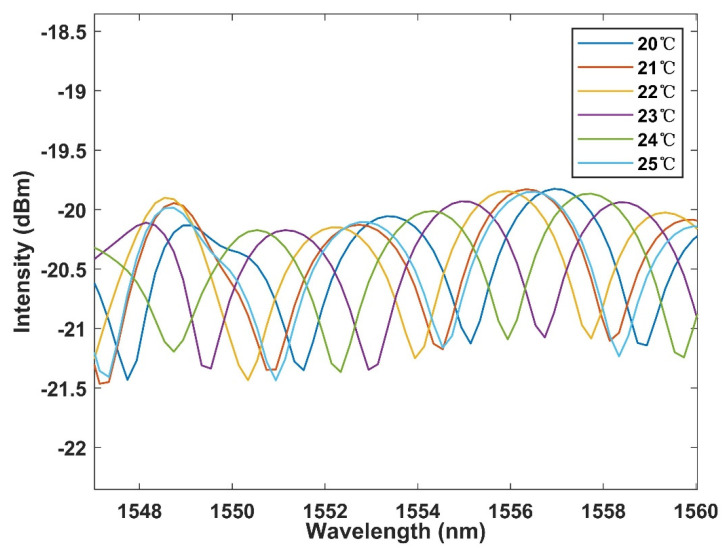
Spectral output characteristics of single Sagnac ring structure with temperature change.

**Figure 7:**
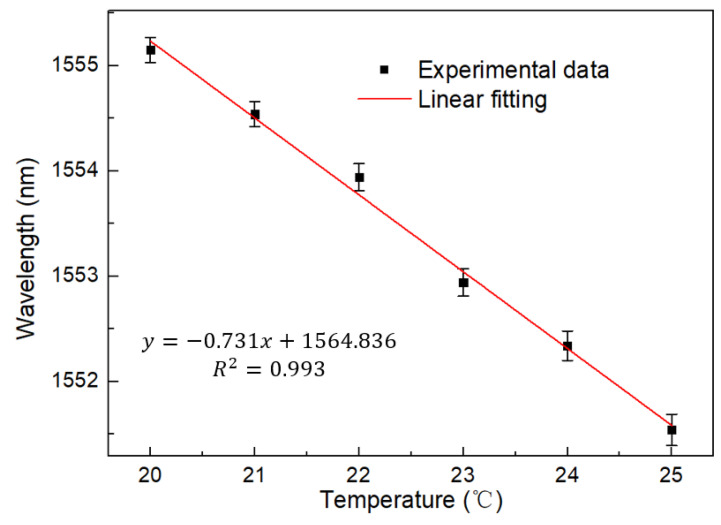
Linear fitting curve of wavelength and temperature of a single Saganc ring at 20–25 °C.

The experiment first demonstrated that the cascaded Sagnac ring was modulated by temperature in the spectral domain. The test results are shown in Figure 6. The wavelength has a blue shift and moves toward the shorter wavelength with the increase of temperature. At the same time, PMF fiber has good temperature response characteristics, so the offset is large. The linear fitting curve is shown in Figure 7, and the temperature sensitivity is up to −0.731 nm/°C. The linearity is 0.993. Good consistency and linear fitting characteristics are maintained. However, compared with other fiber-optic interferometric temperature sensors doped with thermosensitive materials, this does not significantly improve the temperature response.

**Figure 8:**
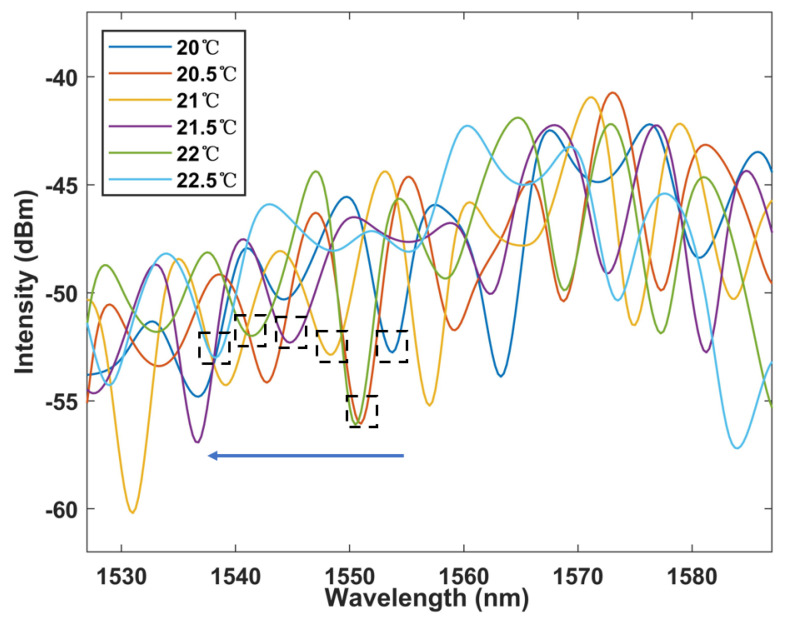
Temperature dependent envelope spectral output characteristics of cascaded Sagnac rings.

**Figure 9:**
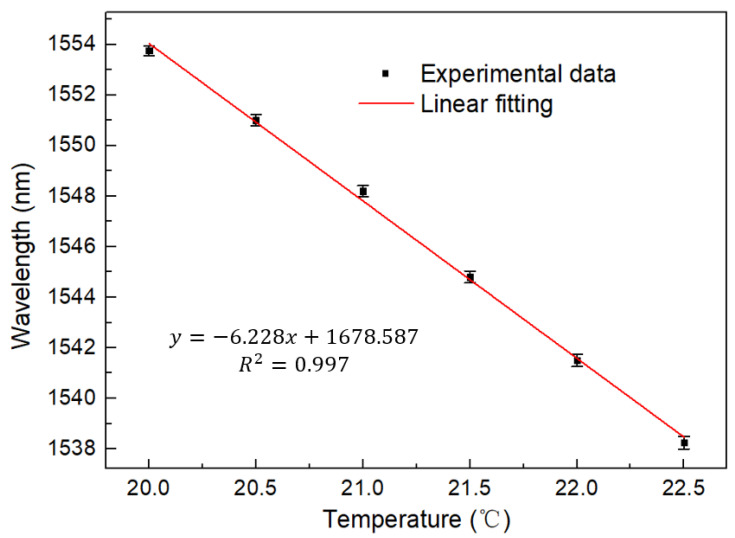
Linear fitting curve of wavelength and temperature of cascaded Saganc rings at 20–22.5 °C.

Cascaded Sagnac rings produce a Vernier effect, and we plotted the spectral envelope as the wavelength response to temperature. As shown in Figure 8, it can be found that the wavelength has a significant blue shift, and the offset is much larger than that of the single loop system. The linear fitting curve is shown in Figure 9, with the sensitivity as high as −6.228 nm/°C, whilst the R square is 0.997. The temperature sensitivity is effectively amplified by 8.5 times.

**Figure 10:**
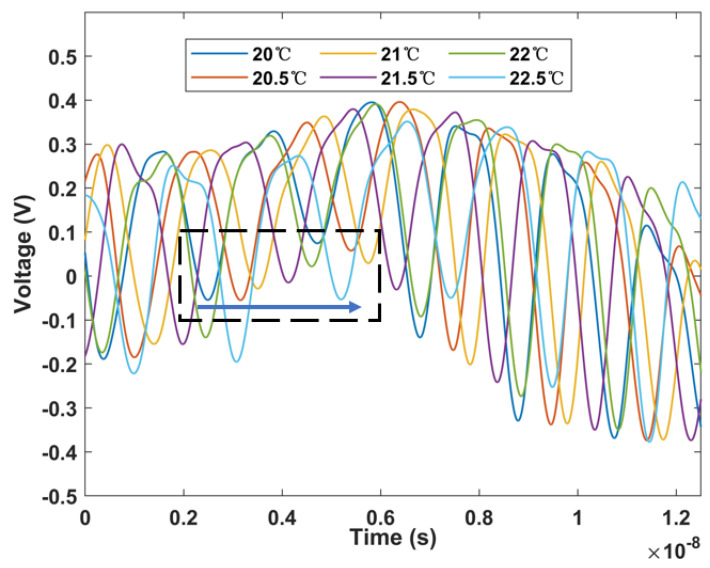
Temporal waveforms characteristics of a single Sagnac ring structure with temperature change.

**Figure 11:**
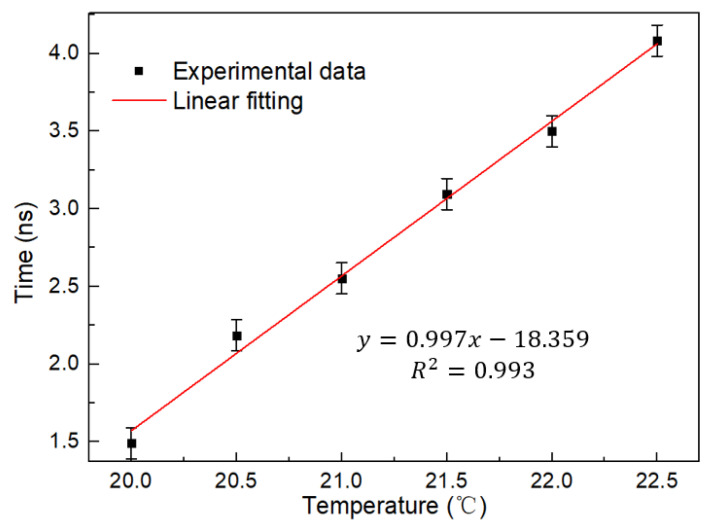
Linear fitting curve of temporal waveform and temperature ofa a single Saganc ring at 20–22.5 °C.

The mapping waveform in the time domain is shown in Figure 10. Since the dispersion coefficient of the dispersive fiber is negative, the shift in the time domain is opposite to the wavelength shift. As the temperature rises, the time moves forward and the pulse repetition frequency is 50 MHz. The linear fitting curve is shown in Figure 11, where it can be seen that the temperature change monitoring in the time domain of 0.997 ns/°C has been successfully realized. This is several orders of magnitude faster than traditional OSA demodulation.

**Figure 12:**
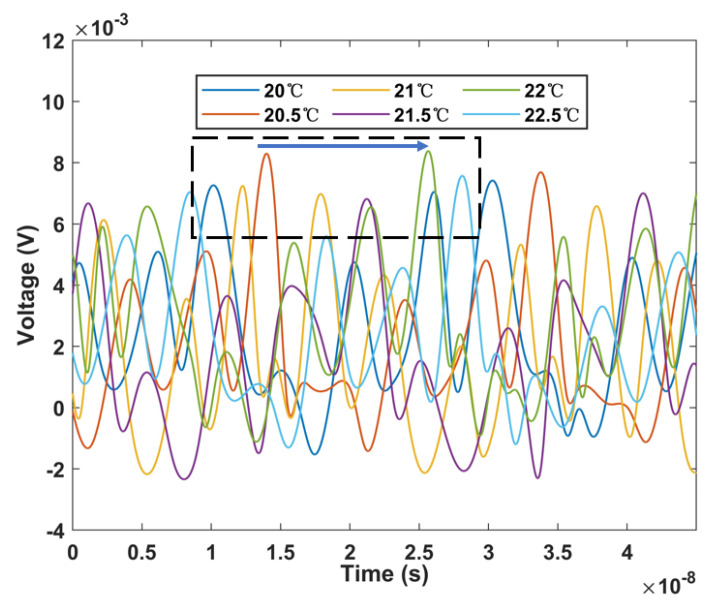
Envelop temporal waveforms characteristics of cascaded Sagnac ring structures with temperature change.

**Figure 13:**
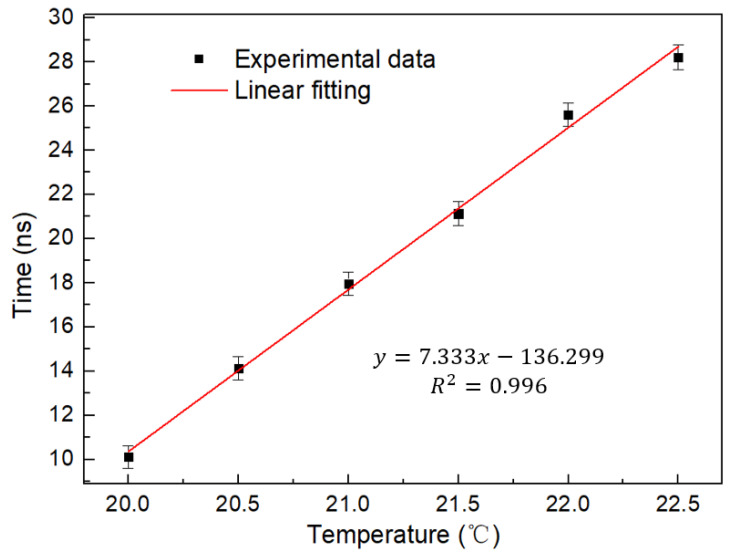
Linear fitting curve of temporal waveform and temperature of cascaded Saganc rings at 20–22.5 °C.

Figure 12 shows the shift of the time domain envelope with temperature. It can be found that compared with the single loop, the shift is significantly increased, the response sensitivity is significantly improved, and the time still keeps moving forward. As shown in Figure 13, its linearity is 0.996, which guarantees its good linear response as a sensor. At the same time, the sensitivity is 7.333 ns/°C, which has effectively improved by 7.4 times. It is more effective in detecting the response to small range temperature changes because of its higher sensitivity. As shown in Figure 13, its error bar is less than 0.13 ns in the range of 20–22.5 °C. This data is repeated five times by us in the time range of 2 h. Therefore, it has good repeatability and stability. Table 1 shows that our sensor has superior temperature sensitivity.

## 4. Conclusions

In conclusion, we successfully realized the optical Vernier effect by cascading Sagnac rings and furthermore improved the temperature sensitivity to −6.228 nm/°C, which is 8.5 times larger than the single ring structure. The Sagnac ring was composed of PMF, which is significantly temperature modulated. As well as this, we also introduced the time stretch effect, which maps the wavelength modulation of the pulsed light to the time domain through the dispersive fiber, because the repetition frequency of the pulsed laser was as high as 50 MHz. Therefore, we have achieved ultra-fast temperature demodulation, with a sensitivity of 7.333 ns/°C, which is 7.4 times larger than that of a single loop, which itself plays an irreplaceable and key role in small range temperature monitoring. This technology is expected to successfully realize real-time online monitoring of temperature and play a key role in detecting rapid ambient temperature changes.

## Figures and Tables

**Table 1:** The sensitivity comparation with other with other temperature sensors based on Sagnac rings.

Structures	Tilt Angle	Refs.
PANDA fiber	−1.46 nm/°C	[39]
Fiber ring laser	−1.739 nm/°C	[40]
TCF-PMF	−1.54 nm/°C	[41]
P-D fiber	−1.804 nm/°C	[42]
FBG cascaded PMF	−1.466 nm/°C	[43]
Current work	−6.228 nm/°C	

## Data Availability

Not applicable.
